# Mycobacteria Treatment Inhibits Bladder Cancer Cell Migration, Invasion, and Anchorage-Independent Growth

**DOI:** 10.3390/ijms252312997

**Published:** 2024-12-03

**Authors:** Marc Bach-Griera, Alba Hernández, Esther Julián

**Affiliations:** 1 Microbiology Unit, Department of Genetics and Microbiology, Biosciences School, Universitat Autonoma de Barcelona, 08193 Bellaterra, Spain; marcbg86@gmail.com; 2 Genetics Unit, Department of Genetics and Microbiology, Biosciences School, Universitat Autonoma de Barcelona, 08193 Bellaterra, Spain; alba.hernandez@uab.cat

**Keywords:** BCG, non-muscle invasive, bacteriotherapy

## Abstract

Bladder cancer (BC) is a highly recurrent and invasive malignancy, with *Mycobacterium bovis* BCG serving as the primary immunotherapy, particularly for non-muscle-invasive bladder cancer (NMIBC). However, the mechanisms underlying BCG’s antitumor effects and the potential of non-tuberculous mycobacteria like *Mycobacterium brumae* remain unclear. This study investigates the antitumor effects of *M. bovis* BCG and *M. brumae* on BC cell migration, invasion, and anchorage-independent growth. BC cell lines representing different stages of tumor differentiation were treated with either *M. bovis* BCG or *M. brumae*. Cell migration was assessed through wound healing and transwell assays, invasiveness by transwell invasion assays, MMP-9 production by gelatin zymography, and anchorage-independent growth via soft agar colony formation. Both mycobacteria inhibited individual cell migration across all BC lines, while collective migration was only reduced in intermediate-grade cells. Both treatments also reduced invasiveness, associated with decreased MMP-9 production. Furthermore, *M. brumae* inhibited anchorage-independent growth across all BC lines, while *M. bovis* BCG had a more selective effect, primarily inhibiting growth in high-grade cells. In conclusion, both mycobacteria reduce migration, invasion, and anchorage-independent growth of BC cells, with their effectiveness varying by species and tumor differentiation grade.

## 1. Introduction

Bladder cancer (BC) is the ninth most common type of cancer worldwide. However, in countries of southwestern Europe and North America, its high incidence rates place it as the fifth most common malignancy [1]. Non-muscle invasive bladder cancer (NMIBC) is the most prevalent form of BC, accounting for approximately 80% of diagnosed cases [2]. The routine management of NMIBC involves transurethral resection of the bladder tumor, followed by weekly intravesical instillations of *Mycobacterium bovis* bacillus Calmette-Guérin (BCG) for 6 weeks. This initial schedule is followed by a maintenance schedule of BCG instillations for 1 year in intermediate-risk patients, or 3 years in high-risk patients, to reduce recurrence and progression rates [3,4,5,6]. Although the exact mechanism of BCG immunotherapy is not fully understood, it is known that BCG attaches to urothelial BC cells via fibronectin and integrin α5β1, leading to internalization through macropinocytosis [7]. Several in vitro studies have demonstrated that mycobacterial infection inhibits the proliferation of BC cells [8,9,10,11,12]. Additionally, infected BC cells behave as non-professional antigen-presenting cells, triggering the massive recruitment of various immune cell populations, including macrophages, dendritic cells, CD4+ and CD8+ lymphocytes, and NK cells [13,14]. The subsequent immune response, involving the release of cytokines and chemokines within the bladder, exerts an effective antitumor effect [15].

While BCG therapy is effective in reducing recurrence and progression of the disease, it fails in a percentage of patients. It is estimated that around 15% of muscle-invasive BC (MIBC) cases have a prior history of NMIBC [16]. Specifically, the risk of progression from NMIBC to MIBC in high-risk NMIBC patients is up to 50% [17,18]. Moreover, *M. bovis* BCG treatment is related to adverse events in a significant proportion of NMIBC patients [19]. Due to these concerns, more effective and safer alternative treatments are being explored. In previous studies conducted by our group, the tumor growth inhibition and immunomodulatory potential of the safe *Mycobacterium brumae* were extensively demonstrated in vitro, ex vivo, and in vivo experiments [9,10,20,21,22,23], making it an interesting comparative tool to study the antitumor effect of *M. bovis* BCG and a potential alternative treatment for NMIBC.

Despite the efficacy of *M. bovis* BCG in preventing BC progression, there is a lack of studies examining the biological phenomena related to the migration and invasiveness of bladder tumor cells following BCG treatment. Garden et al. [24] demonstrated that *M. bovis* BCG inhibits migration and invasion by using the EJ BC cell line, a derivative from the T24 BC cell line [25]. Furthermore, the effect of *M. brumae* on BC cell migration and invasion remains unknown. Cellular migration and invasion are critical hallmarks of metastasis and are directly linked to the epithelial-mesenchymal transition (EMT) [26,27]. EMT increases the motility and invasiveness of tumor cells, involving a shift from an epithelial to a mesenchymal phenotype. This transition enables the degradation of the surrounding extracellular matrix (ECM) through the upregulation and secretion of active matrix metalloproteinases (MMPs), which are crucial in the intravasation and dissemination of neoplastic cells and are closely related to the progression of NMIBC to MIBC [28,29,30,31,32,33].

To unravel the possible role of *M. bovis* BCG and *M. brumae* in inhibiting BC migration and invasion, we study here five different BC cell lines, covering different malignance grades, to assess the role of mycobacteria in these phenotypical processes using four different in vitro experimental models: wound healing assay, transwell migration and invasion assays, and soft-agar colony formation assay.

## 2. Results

### 2.1. BCG and M. brumae Infections Decrease Collective Migration Capability in Intermediate-Grade BC Cells

**Figure 1:**
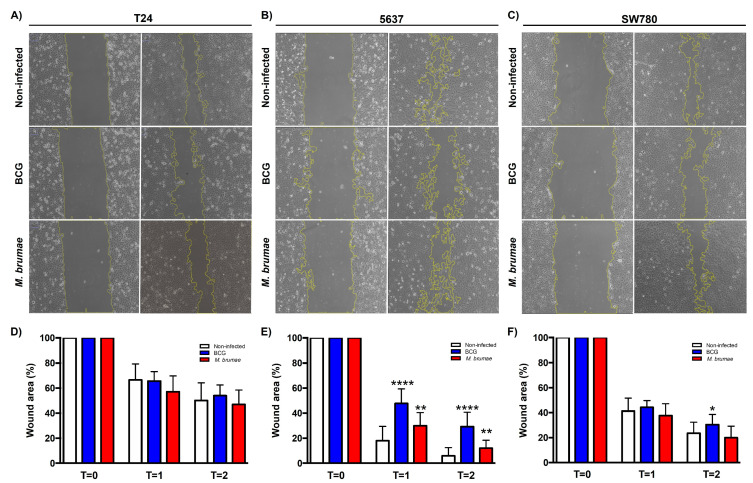
Effect of BCG and *M. brumae* on wound healing of BC cells. BCG infection significantly reduces cell migration in both the 5637 and SW780 BC cell lines, while *M. brumae* infection significantly decreases migration in the 5637 cell line. One representative experiment is shown for T24 (**A**), 5637 (**B**), and SW780 (**C**) cell lines at T = 0 and T = 2. The scale of the images is comparable. Graphs show percentage of occupied area by non-infected (control) and mycobacteria-infected cells at different time points (T = 0, 0 h; T = 1, 16 h; T = 2, 24 h) for T24 (**D**), 5637 (**E**), and SW780 (**F**) cell lines. Data represent the means ± SD from three independent experiments. Statistical comparisons were performed using Student’s *t*-test. **** *p* < 0.0001; ** *p* < 0.01; * *p* < 0.05 versus non-infected cells.

Wound healing assay reveals that mycobacteria infection affects collective BC cell migration depending on the differentiation grade of each tumor cell line. No significant differences were observed in high-grade T24 cells between infected and non-infected cells (Figure 1A and Supplementary File). However, statistically significant differences were seen in intermediate-grade 5637 and low-grade SW780 cells when they were infected with mycobacteria (Figure 1B,C and Supplementary File). In the 5637 cell line, BCG- and *M. brumae*-infected cells exhibited wound closure rates of 70.8% and 87.9%, respectively, compared to 94.1% in non-infected cells by the experiment’s end (T = 2, Figure 1B). In the low-grade SW780 cells, BCG infection resulted in a significantly lower wound closure rate (65.2%) compared to non-infected cells, while the rate was similar between *M. brumae*-infected and non-infected cells, showing fewer cell-free areas (Figure 1C).

### 2.2. Mycobacteria Infection Decreases the Single Cell Migration Capability of BC Cells

**Figure 2:**
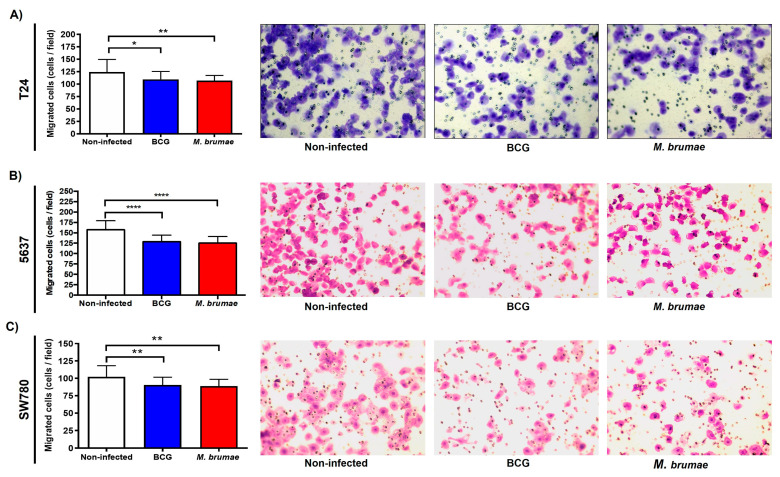
Single-cell migration using the transwell migration assay after mycobacteria infection. Transwell migration experiments demonstrate significant inhibition of cell migration in mycobacteria-infected T24, 5637, and SW780 BC cell lines. Graphs show cell counts per field (N = 20, obtained from five different random fields *per transwell*) for T24 (**A**), 5637 (**B**), and SW780 (**C**) cell lines, along with representative images for each condition. The scale of the images is comparable (at ×100 magnification). Data are presented as means ± SD from four independent experiments. Statistical comparisons were made using one-way analysis of variance (ANOVA) followed by Dunnett’s post-hoc test: **** *p* < 0.0001; ** *p* < 0.01; * *p* < 0.05 versus non-infected cells.

As Figure 2 shows, both mycobacteria treatments had a modest though significant inhibitory effect on single cell migration in all BC cells studied compared to non-infected BC cells (and Supplementary File). Fewer infected cells were able to migrate and attach to the lower surface of the transwell inserts, as seen in representative pictures of each condition of Figure 2. When migration was expressed as a percentage relative to non-infected cells, *M. brumae* and BCG infection reduced migration by 13.5% and 9.7%, respectively, in T24 cells. For 5637 cells, the migration rates diminished by 19.3% for *M. brumae* infection and 19% for BCG with respect to non-infected cells, while *M. brumae* and BCG infection reduced migration by 12.7% and 11.4%, respectively, for SW780 cells. No significant differences were found between the two mycobacterial treatments.

### 2.3. M. brumae Treatment Alone Decreases Anchorage-Independent Growth in BC Cell Lines

**Figure 3:**
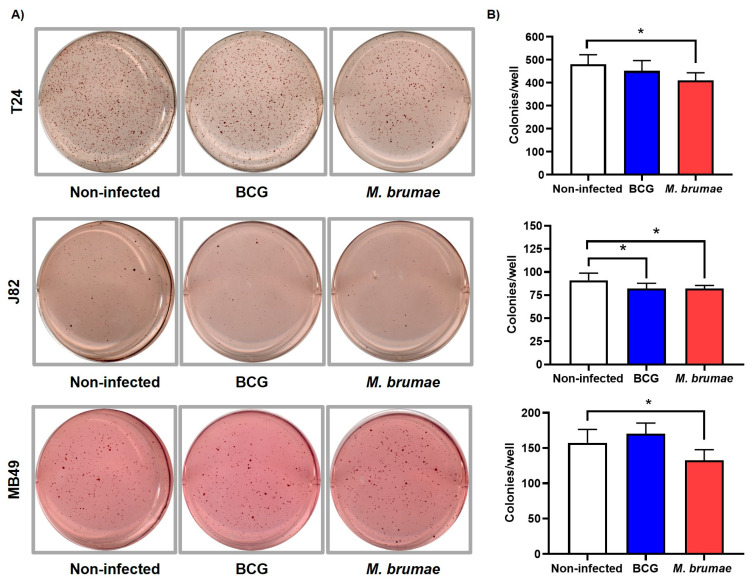
Evaluation of anchorage-independent growth using the soft agar colony formation assay. The soft agar colony formation assay reveals statistically significant differences in the anchorage-independent growth capacity of BC cell lines infected with *M. brumae*. Representative images of colony formation (**A**) and colony counts (**B**) in non-infected, BCG-, and *M. brumae*-infected T24, J82, and MB49 BC cells in soft agar. Data represent the means ± SD from three independent experiments. Statistical comparisons were performed using one-way analysis of variance (ANOVA) followed by Dunnett’s post-hoc test: * *p* < 0.05; versus non-infected cells.

To study the anchorage-independent growth of non-infected and mycobacteria-infected cells, human intermediate-grade (5637) and high-grade (T24 and J82) cells were initially selected. Experiments with intermediate-grade 5637 cells showed no colony formation in either infected or non-infected conditions, as expected. Thus, to increase the number of studied cell lines, the murine high-grade MB49 cell line was included. The MB49 cell line is extensively used to study the murine orthotopic model of the disease. As shown in Figure 3A,B, *M. brumae* infection significantly reduced anchorage-independent growth (colony formation) in all BC cell lines, with reductions of 15.5%, 12.3%, and 17.5% for T24, J82, and MB49 cells, respectively, compared to non-infected cells. In contrast, BCG treatment only produced statistically significant differences in the J82 cell line, where a 12.1% reduction in colony formation was observed (Figure 3 and Supplementary File).

### 2.4. M. brumae and BCG Treatments Decrease Cell Invasion by Inhibiting Active MMP-9 Secretion

**Figure 4:**
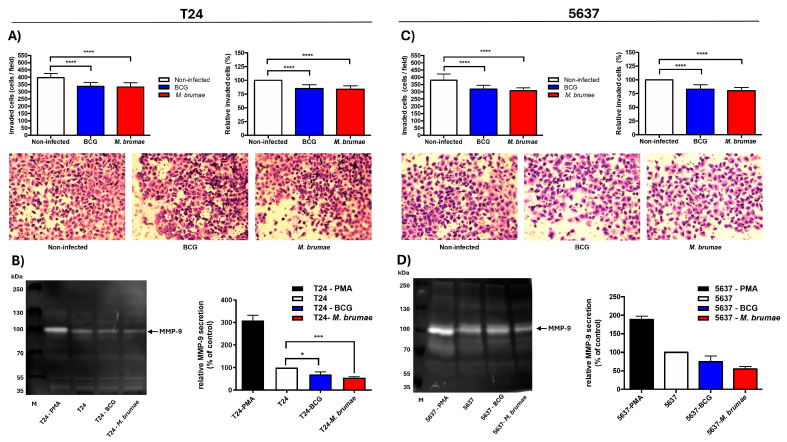
Effect of mycobacteria treatment on BC cell invasion and MMP production. Mycobacteria-infected BC cells show lower cell counts and reduced MMP-9 activity in the invasion cell assay compared to untreated cells. Cell counts of invaded cells, relative invasion percentages, and representative images of invasion assays in T24 (**A**) and 5637 (**C**) cell lines. The scale of the images is comparable (at ×100 magnification). Representative gelatin zymography and relative MMP-9 secretion in T24 (**B**) and 5637 (**D**) cells. Cell invasion assay results are presented as means ± SD from three independent experiments. MMP-9 secretion graphs represent means ± SD from two independent experiments. Statistical comparisons were performed using one-way analysis of variance (ANOVA) followed by Dunnett’s post-hoc test. **** *p* < 0.0001; *** *p* < 0.001; * *p* < 0.05 versus non-infected cells.

When transwell cell invasion assay was performed, a strong statistical reduction in cell counts was found in mycobacteria-infected compared to non-infected BC cells (Figure 4). Both T24 and 5637 cell lines showed a similar response to each mycobacterial treatment. BCG treatment reduced invasion by 14.9% in T24 cells and 17.2% in 5637 cells, while *M. brumae* treatment reduced invasion by 16.2% and 19.6%, respectively (Figure 4A,C and Supplementary File).

As gelatine zymography shows, a band corresponding to MMP-9 (92-KDa) activity was observed in the positive control, that is, conditioned media collected from PMA-treated cells (Figure 4B,D). In conditioned media from BCG- and *M. brumae*-infected T24 cells, the intensity of the MMP-9 bands was significantly reduced compared to that obtained from non-infected cells (Figure 4B). For 5637 cells, MMP-9 activity in mycobacteria-infected conditioned media was also lower than in that from non-infected cells, although the difference was not statistically significant (Figure 4D). No bands corresponding to MMP-2 activity (72-kDa) were observed under the analyzed conditions.

## 3. Discussion

In this study, we report that mycobacteria treatment exerts an anti-oncogenic effect on tumor migration and progression, depending on the specific mycobacterium used and the differentiation status of bladder cancer (BC) cells. Our aim was to investigate whether direct contact between *M. bovis* BCG and BC cells mimics its known effectiveness in reducing progression in NMIBC patients. We focused on evaluating, through in vitro studies, the role of *M. bovis* BCG treatment in various biological processes involved in the invasiveness and migration of BC cells. These processes are key mechanisms for tumor dissemination, as described by Hanahan and Weinberg in their publication on the hallmarks of cancer [34]. Our study has examined both cell migration and invasion, anchorage-independent tumor growth, and active MMP-9 production (via gelatin zymography) using BCG immunotherapy compared to another mycobacterium-based antitumor agent. Although previous studies have demonstrated the ability of *M. brumae* to inhibit tumor cell growth in vitro and in vivo [9,10,20,21,22,23], the impact on BC cell migration and invasion using a live non-pathogenic mycobacterium such as *M. brumae* has not been reported before.

Our first key finding is the decreased migratory capability of BC cells following infection with *M. bovis* BCG and *M. brumae* compared to non-infected cells. Cell migration, whether individual or collective, is essential for wound healing and tissue homeostasis. However, migration also plays a critical role in tumor formation and dissemination, including in BC [35,36]. In this study, individual and collective cell migration were evaluated using inserts assay and wound healing assay, respectively, to assess BC cells that have undergone EMT for dissemination across the basement membrane and into the surrounding stroma [37,38]. The results show that while individual cell migration was reduced in all mycobacteria-treated cells, collective cell migration was significantly reduced only in intermediate-grade BC cells. Specifically, single-cell motility was inhibited in all three bladder tumor cell lines following treatment with both *M. bovis* BCG and *M. brumae* (Figure 4A,B). These findings align with those of Garden et al. [24], who observed reduced migration in *M. bovis* BCG-treated EJ human transitional carcinoma cells. The authors demonstrated that the fibronectin attachment protein (FAP) of the *M. bovis* BCG TICE strain binds to fibronectin or integrin α5β1 receptors on the surface of bladder tumor cells [13,39], interfering with cell migration. The role of fibronectin in promoting growth, survival, and invasion of cancer cells has been highlighted by in vitro studies. *M. brumae* is also known to bind fibronectin [20], which may explain our results. Unlike individual migration, T24 cells continued to migrate collectively even after mycobacteria infection (Figure 2A), whereas 5637 and SW780 cells exhibited inhibited collective migration. This difference might be due to the greater ability of high-grade BC cells like T24 to internalize *M. bovis* BCG compared to lower-grade BC cells [7,40]. In our experimental conditions, we observed similar internalization rates (about 10% of infected cells) for both *M. bovis* BCG and *M. brumae* in T24 cells. Higher intracellular mycobacteria levels might influence cell characteristics by disrupting cell-cell interactions and thus motility. While studies of mycobacteria-related interference of invasion are scarce, a reduction in cellular proliferation, angiogenic factors, and both cell migration and invasion in *M. bovis* BCG-treated head and neck cancer cells, downregulating the expression of MMPs [41,42], has been demonstrated, suggesting its potential role as antitumor agent in this type of cancer.

In a second set of experiments, we performed transwell invasion assays to evaluate the invasive capacity of malignant cells. Invasiveness refers to cancer cells’ ability to disrupt cell-cell junctions and reorganize the extracellular matrix (ECM), facilitating the spreading of tumor cells into the surrounding tissues and the entrance in vessels lumens to colonize distant organs [43]. Our results showed that T24 and 5637 cell invasiveness was reduced after treatment with either *M. bovis* BCG or *M. brumae*. In this regard, Tsui et al. correlated the production of IL-6 with reduced cell invasiveness in the T24 tumor cell line [44], suggesting that IL-6 modulates EMT by upregulating E-cadherin (associated with epithelial characteristics) and downregulating N-cadherin and vimentin (markers of invasive cells). Both *M. bovis* BCG and *M. brumae* treatments stimulate IL-6 production in BC cells [6,7,9,24,31], which may be related to their ability to inhibit BC cell proliferation [45] and potentially influence migration.

To gain insight into the underlying mechanism by which mycobacteria inhibit BC cell invasiveness, the production of MMP-9 was evaluated. MMPs are gelatinases that degrade the ECM, which is crucial to maintaining the physiological microenvironment and homeostasis. Disruption of this balance can promote tumor growth, invasion, and metastasis [46]. As mentioned, the integrity of the extracellular matrix is essential for the maintenance of epithelial cells, and during cell invasiveness processes resulting from EMT, there is an increase in the production of certain MMPs [47]. Our zymography results showed reduced MMP-9 production in mycobacteria-treated BC cells compared to non-infected cells. In contrast, Kageyama et al. [48] did not detect MMP production in *M. bovis* BCG (Tokio strain)-treated BC cells, which may be due to methodological differences. Our study cultured BC cells in serum-free medium for 72 h to condition their metabolism, enabling evaluation of the expression of active MMPs. Our findings are consistent with studies of BCG-treated head and neck squamous carcinoma cells, which also showed reduced MMP-9 expression following infection with *M. bovis* BCG [42]. This MMP inhibition may be linked to reduced telomerase activity in BC cells following *M. bovis* BCG infection [49]. Additionally, related to non-tuberculous mycobacteria, heat-killed *Mycobacterium indicus pranii* has been shown to inhibit melanoma cell invasion by reducing MMP-9 expression [50]. To our knowledge, our study is the first to demonstrate the anti-invasive capacity of a live, non-pathogenic mycobacterium like *M. brumae*.

Metastasis is a complex process involving tumor cell infiltration of surrounding tissue, entry into blood vessels (intravasation), survival in circulation, and colonization of distant organs [51]. One characteristic of tumoral cells, when they are detached or misplaced from their original location, is the resistance to avoid the self-strategy of programmed cell death, known as anoikis in non-tumoral cells [52]. Therefore, the proliferation of these cells is dependent on anchorage to an extracellular matrix [53]. In our study, the ability of tumor cells to grow independently of the ECM was assessed using a soft agar colony formation assay. Our results showed a decrease in the number and size of BC cells treated with *M. brumae*, whereas *M. bovis* BCG significantly reduced anchorage-independent growth only in the J82 cell line. Related to this finding, a recent study by Mahe and colleagues identified a positive feedback loop between the expression of the FGFR3 receptor, a tyrosine kinase receptor strongly associated with the development and progression of BC, and the positive regulation of MYC proto-oncogene mRNA levels, thereby promoting anchorage-independent growth in bladder tumor cells [54]. *M. bovis* BCG infection reduces FGFR3 expression in BC cells, potentially explaining the reduced anchorage-independent growth observed in our experiments [55]. Further research is needed to determine whether *M. brumae* inhibits colony formation through this or other mechanisms.

## 4. Materials and Methods

### 4.1. Bacterial Strains and Infection Procedure

*M. bovis* BCG Connaught (ATCC 35745) and *M. brumae* (ATCC 51384T) were grown on Middlebrook 7H10 medium (Difco Laboratories, Detroit, MI, USA) supplemented with 10% oleic acid-albumin-dextrose-catalase (OADC) enrichment for 3 weeks and 1 week, respectively, at 37 °C. BCG and *M. brumae* cells were resuspended in sterile phosphate buffered saline (PBS) and adjusted to a 1.0 McFarland turbidity standard as previously described [9,20] to get the required multiplicity of infection (MOI) for each experiment. To confirm the dose of each experiment, representative mycobacterial suspensions of each treatment were serially diluted in PBS, and CFU were counted after plating on Middlebrook 7H10 medium as previously described [56].

### 4.2. Cell Culture Conditions

Murine (MB49) or human (T24, SW780, 5637, and J82) urinary BC cells lines were maintained in Dulbecco’s Modified Eagle Medium/Nutrient Mixture (DMEM) (GIBCO, Auckland, NZ, USA) or DMEM-F12 (GIBCO), respectively, which were supplemented with 10% heat-inactivated fetal bovine serum (FBS) (HyClone, Northumberland, UK) plus penicillin (10,000 U/mL, Laboratories ERN, Barcelona, Spain) and streptomycin (10 mg/mL, Laboratories Reig Jofre, Barcelona, Spain) (complete medium, CM). Cells were always incubated at 37 °C in a 5% CO_2_ humidified atmosphere [9].

### 4.3. Collective Cell Migration Through Wound-Healing Assay

To carry out the wound-healing assay, T24, 5637, and SW780 BC cells were seeded into 24-well cell culture plates (Nunclon, Roskilde, Denmark) at 2 × 10^5^ cells/well or 3 × 10^5^ cells/well for SW780 in CM. After reaching a confluence monolayer, cells were infected at MOI 10 with BCG or *M. brumae* or left untreated. After 3 h of incubation, an artificial wound was created using a sterile 10 µL pipette tip, and cells were washed five times with PBS. CM without FBS for T24 and 5637 cells or CM with 1% of FBS for SW780 cell line was added to evaluate cell migration. These optimized conditions using serum starvation were considered the gold standard for comparison purposes. Previous studies in our laboratory showed that the addition of mitomycin C before mycobacteria infection has a synergic effect in growth inhibition rates, which could alter the interpretation of the true migration of mycobacteria-treated cultures. The plates were incubated at 37 °C with 5% CO_2_, monitored, and photographed using an inverted microscopy Axio Observer A1 (Zeiss, Oberkochen, Germany) at different time points: 0, 12, and 24 h. The wound-healing area was calculated as the percentage area of the initial wound with respect to the percentage area of wound closure at 24 h using the ImageJ ver.2.0.0 software and the MRI Wound Healing Tool macro (http://dev.mri.cnrs.fr/projects/imagej-macros/wiki/Wound_Healing_Tool (accessed on 1 October 2022)) [57,58,59].

### 4.4. Single Cell Migration and Invasion Through Transwells Assays

Migration and invasion assays were performed using transwell inserts (8 µm pore size) (Falcon, Schaumburg, IL, USA) as described previously with slight modifications [60,61]. Briefly, T24, 5637, and SW780 cells, with a previous starving of 24 h, were seeded into 24-well cell culture plate (Nunclon, Denmark) at 2 × 10^5^ cells/mL in CM without antibiotics. After 3 h of incubation, cells were infected with BCG or *M. brumae* (MOI 10:1) as mentioned above. Three hours later, the cells were washed five times with warm PBS and detached with 0.2 mL of trypsin (Biowest, Nuaillé, France). The treated cells were collected by centrifugation at 250× *g* for 10 min, and the pellets were resuspended in DMEM-F12 medium. For the migration assay, 1.5 × 10^4^ cells (for T24 and 5637) or 3 × 10^4^ cells (for SW780 BC cells) in 0.2 mL of CM without FBS were added into the transwell inserts. To perform the invasion assay, 3 × 10^4^ mycobacteria-infected T24 and 5637 cells in 0.2 mL of CM without FBS were seeded in transwell inserts precoated with 3 mg/mL of Matrigel (Corning, New York, NY, USA). Then, 0.8 mL of CM was added into the wells of 24-well cell culture plates (Falcon, USA) as chemoattractant media. After 24h of incubation, migrated cells were fixed with methanol solution and stained with 0.2% crystal violet (Sigma Aldrich, St. Louis, MO, USA). Five random different fields of each stained transwell inserts were photographed under a microscope (Nikon Eclipse TE-2000·E, Tokyo, Japan) and counted using the ImageJ ver.2.0.0 software.

### 4.5. Anchorage Independent Growth by Soft Agar Colony Formation Assay

To evaluate anchorage independent growth, a bottom layer of CM with 0.6% bacteriological agar (Scharlab, Barcelona, Spain) was set in 6 well-culture plates (Nunclon, Denmark). The upper layer consisted of a single-cell suspension of non-infected and BCG- or *M. brumae*-infected (MOI 10:1) T24, J82, and MB49 cells (2.5 × 10^3^ cells) with 0.3% bacteriological agar. After incubation for 3 weeks (T24 and MB49 BC cell lines) or 4 weeks (J82 BC cell line) at 37 °C with 5% CO_2_, grown cell colonies were stained adding 1 mL (1 mg/mL) of 2-(4-Iodophenyl)-3-(4-nitrophenyl)-5-phenyl-2H-tetrazolium chloride (INT) (Sigma-Aldrich, St. Louis, MO, USA) on the wells and incubating them for 18–24 h [62]. Images of the plates were then taken using a CanoScan LiDE 200 scanner (Canon, Tokyo, Japan), and colonies > 50 µm diameter, of each condition, were counted using the Open CFU ver.3.9 software tool.

### 4.6. Secretion of Matrix Metalloproteinases (MMPs) by Zymography

For measuring the activity of secreted matrix metalloproteinases (MMPs), conditioned media were obtained from mycobacteria-infected and non-infected T24 and 5637 cells incubated for 72 h in FBS-free medium. In parallel, T24 and 5637 BC cells were incubated for 72 h in FBS-free medium plus phorbol 12-myristate 13-acetate (100 ng/mL) (PMA, Abcam, Cambridge, UK) as a positive control for secretion of MMPs [63]. All conditioned media were collected, and the activity of secreted MMP was measured using gelatin zymography. Briefly, 40 µL of each sample were loaded in 7.5% SDS-PAGE gels containing gelatin (1 mg/mL) at 150 V for 90 min and then incubated overnight in 1× zymogram renaturing buffer (BioRad, Hercules, CA, USA). Gels were later incubated overnight again with 1× zymogram developing buffer (BioRad) at 37 °C to achieve maximum sensitivity. Gels were then stained with 0.1% of Coomassie brilliant blue R-250 (BioRad) for 1 h and de-stained in methanol/acetic acid/distilled H_2_O solution (4:1:5) until the areas of gelatinase activity appeared as clear bands [64]. Gel zymograms were photographed using Chemidox XRS+ system (BioRad), and the intensity of the bands was then analyzed by plotting a curve and calculating the area under curve through ImageJ ver.2.0.0 software [65].

### 4.7. Statistical Analyses

All assays were repeated three times with three biological replicates each. Data were represented as arithmetic means ± SD. Statistical comparisons were performed using Student’s *t*-test and the Mann-Whitney U test or one-way analysis of variance (ANOVA) followed by Dunnett’s post-hoc test as indicated in the figure legends. Statistical significance was defined as a *p*-value < 0.05. Statistical analysis and graphics were done with the program GraphPad Prism 8.0 (GraphPad Software Inc., San Diego, CA, USA) program.

## 5. Conclusions

In summary, our results demonstrate that both *M. bovis* BCG and *M. brumae* exert antitumor effects on BC cells by reducing cell migration and invasion in in vitro models. These findings broaden the differential mycobacterium-driven anti-invasive effect on BC cells related to anchorage independent growth, suggesting that there is room for improvement of the *M. bovis* BCG antitumor capacity and warranting further studies to consolidate non-tuberculous mycobacterium-triggered antitumor effects.

## Data Availability

The original data presented in the study are openly available as Supplementary File.

## Supplementary Materials

The following supporting information can be downloaded at https://www.mdpi.com/article/10.3390/ijms252312997/s1.
